# Matrix factorization reveals aging-specific co-expression gene modules in the fat and muscle tissues in nonhuman primates

**DOI:** 10.1038/srep34335

**Published:** 2016-10-05

**Authors:** Yongcui Wang, Weiling Zhao, Xiaobo Zhou

**Affiliations:** 1 Center for Bioinformatics & Systems Biology, Department of Radiology, Wake Forest School of Medicine, Winston Salem, NC, USA; 2 Key Laboratory of Adaptation and Evolution of Plateau Biota, Northwest Institute of Plateau Biology, Chinese Academy of Sciences, Xining, China

## Abstract

Accurate identification of coherent transcriptional modules (subnetworks) in adipose and muscle tissues is important for revealing the related mechanisms and co-regulated pathways involved in the development of aging-related diseases. Here, we proposed a systematically computational approach, called ICEGM, to Identify the Co-Expression Gene Modules through a novel mathematical framework of Higher-Order Generalized Singular Value Decomposition (HO-GSVD). ICEGM was applied on the adipose, and heart and skeletal muscle tissues in old and young female African green vervet monkeys. The genes associated with the development of inflammation, cardiovascular and skeletal disorder diseases, and cancer were revealed by the ICEGM. Meanwhile, genes in the ICEGM modules were also enriched in the adipocytes, smooth muscle cells, cardiac myocytes, and immune cells. Comprehensive disease annotation and canonical pathway analysis indicated that immune cells, adipocytes, cardiomyocytes, and smooth muscle cells played a synergistic role in cardiac and physical functions in the aged monkeys by regulation of the biological processes associated with metabolism, inflammation, and atherosclerosis. In conclusion, the ICEGM provides an efficiently systematic framework for decoding the co-expression gene modules in multiple tissues. Analysis of genes in the ICEGM module yielded important insights on the cooperative role of multiple tissues in the development of diseases.

Previous studies on aging have indicated that fat, and heart and skeletal muscle tissues are involved in the longevity and age-related metabolic dysfunction^1^,^2^,^3^,^4^,^5^. Studies of longer duration are needed for fully understanding the effects of aging on fat and muscles. The rapid development of high-throughput experimental technologies has resulted in the accumulation of genome-scale transcriptional data in multiple conditions (e.g. tissue-types and disease states)^6^,^7^. It provided a great opportunity for exploring the mechanism of disease-specific biological process through analysis of multiple large-scale networks simultaneously and discovery of common pattern^8^,^9^. Thus, the mechanism of the aging effects on the fat and muscles could be explored through identification of the common gene modules (subnetworks) based on the genome-scale transcriptional data in fat and muscle tissues.

Many computational approaches have been used for network inference with a single condition^10^,^11^,^12^. However, only few computationally efficient methods have been used for large-scale network inference across multiple conditions for now. These methods were developed for either identifying the ‘difference’ between diverse networks^13^,^14^ or unveiling the ‘common’ components shared by networks in multiple conditions^15^,^16^,^17^. Complementary to these methods, spectral methods, such as Generalized Singular Value Decomposition (GSVD), have been proposed to investigate the subnetworks across two conditions^18^,^19^. In addition, higher-order GSVD (HO-GSVD), the upgraded version of GSVD was also proposed for the analysis of the networks in more than two conditions^20^,^21^.

For example, Ponnapalli *et al*. applied HO-GSVD to compare multiple datasets with identical columns for the analysis of common structures of multiple datasets^20^. Xiao *et al*. developed a novel algorithmic HO-GSVD for identifying ‘common’ and ‘condition-specific’ network-modules simultaneously across multiple conditions with parameter-free property^21^. Here, to reveal the ‘common’ gene modules shared by fat and muscle tissues, we proposed a novel mathematical framework of HO-GSVD for **I**dentification of the **C**o-**E**xpression **G**ene **M**odules (ICEGM). Specifically, the expressions in multiple tissues were represented by the large-scale matrices. Then these large-scale matrices were decomposed through HO-GSVD with diverse left basis matrices and diagonal matrices, as well as an identical right basis matrix. The column of right basis matrix with significant bimodal distribution v^*^ was chosen to help us identify the co-expression genes. Finally, the co-expression gene was determined through estimating the significance of elements of v^*^ locating in the tail area of a small weight component of the bimodal distribution. The DAVID bioinformatics resources (http://david.abcc.ncifcrf.gov) were introduced to validate the functional connectivity of gene modules, the modules with functional enrichment score (Benjamini adjusted p-value) smaller than 0.05 were remained for further analyses.

ICEGM was validated on the adipose, and heart and skeletal muscle tissues in old and young female African green vervet monkeys. The functional/disease annotation and canonical pathway analysis were performed via IPA platform (http://www.ingenuity.com/products/ipa), while cell-type enrichment analysis was carried out through CTen (Cell Type Enrichment analysis of the microarray data) platform (http://www.influenza-x.org/~jshoemaker/cten/). We uncovered the co-expression gene modules in adipose, heart and skeletal muscle tissues and found the functional connectivity between them. Further analysis indicated that co-expression genes were enriched in the development of inflammation, CVD, and skeletal diseases. Comprehensive disease annotation and analysis of the canonical pathways on the genes identified from ICEGM modules indicated that immune cells, adipocytes, cardiomyocytes, and smooth muscle cells played a synergistic role in the cardiac and physical functions in the aged monkeys by regulation of the biological processes associated with metabolism, inflammation, and atherosclerosis.

## Results

**Figure 1:**
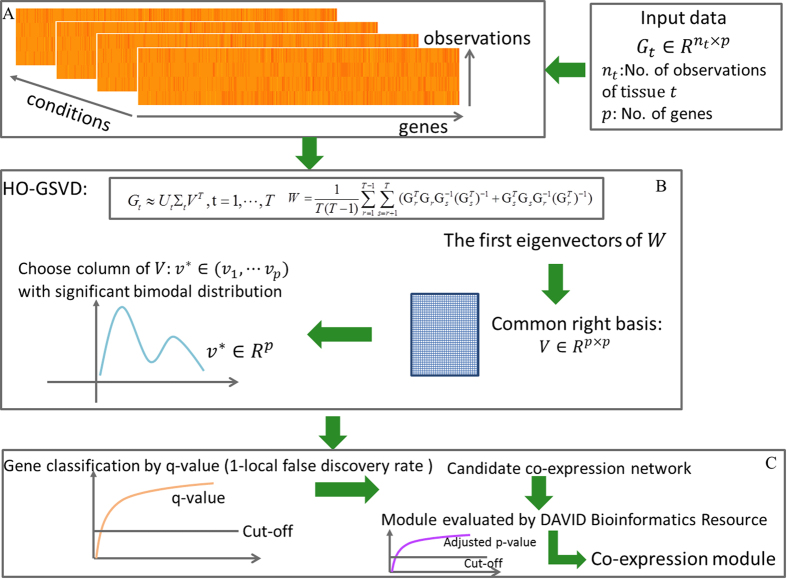
Schematic illustration of the ICEGM. (**A**) Gene expressions in different conditions were represented by large-scale matrices with row as samples and column as genes. (**B**) The multiple matrices were decomposed through HO-GSVD with diverse left basis matrices and diagonal matrices, as well as an identical right basis matrix, and the right basis vector with significant bimodal distribution was calculated. (**C**) The genes significantly located in the tail area of small weight components of bimodal distribution were selected. The gene modules were then generated through the functional enrichment validation via DAVID bioinformatics software.

In this work, we developed a novel method to systematically identify the co-expression gene modules in multiple tissues, called as ICEGM. The schematic illustration of ICEGM is shown in the Fig. 1. It is firstly to arrange the individually expression data as a large-scale matrix with row as samples and column as genes (Fig. 1A). Then the simplified mathematical framework of HO-GSVD was developed to identify the co-expression gene modules in multiple tissues. Specifically, the matrices were decomposed through HO-GSVD with diverse left basis matrices and diagonal matrices, as well as an identical right basis matrix. The right basis matrix were obtained through the decomposition of the input co-expression matrices (Fig. 1B). The column of right basis matrix with the significant bimodal distribution v^*^ was chose to detect the co-expression genes (Fig. 1B). Finally, the gene was chosen as co-expression genes through estimating the significance of elements of v^*^ locating in the tail area of a small weight component of the bimodal distribution (Fig. 1C). The functional enriched modules were unveiled finally through the functional enrichment validation via DAVID bioinformatics software (Fig. 1C).

### The co-expressed gene modules in four types of monkey tissues

We did ICEGM analysis on the microarray data in fat and muscle tissues collected from 11 young and older adults of monkeys. The five right-basis vectors with significant bimodal distribution were obtained, indicating the five potential co-expression gene modules were generated for further analysis. Two of them had functional enrichment scores (Benjamini adjusted p-value) less than 0.05 (Table S1), including the cluster 3 with 28 co-expression genes and cluster 5 with 142 co-expression genes. By cluster-quality (CQ) checking, we found that although cluster 5 had a lower CQ, its significance score was higher (Figure S1) than the cluster 3 did. In addition, the cluster 5 consisted of 142 genes, thus we focused on the cluster 5 in the following analysis.

Figure S2 shows the co-expression gene network for the cluster 5. The functional/disease annotations indicated that the genes in the cluster 5 were associated with the development of cardiovascular disease (CVD), inflammation, skeletal disorders, and cancers. These disease associated genes are grouped and highlighted in the Figure S2, respectively. Figure S2 shows that, most of correlations among the CVD-associated genes are positive, so dose the inflammation-associated genes; while there are both positive and negative relations among skeletal disorder-associated genes, and the cancer-associated genes. These results indicated that the molecular mechanisms for cancer development and skeletal disorder diseases might be more complicated.

**Figure 2:**
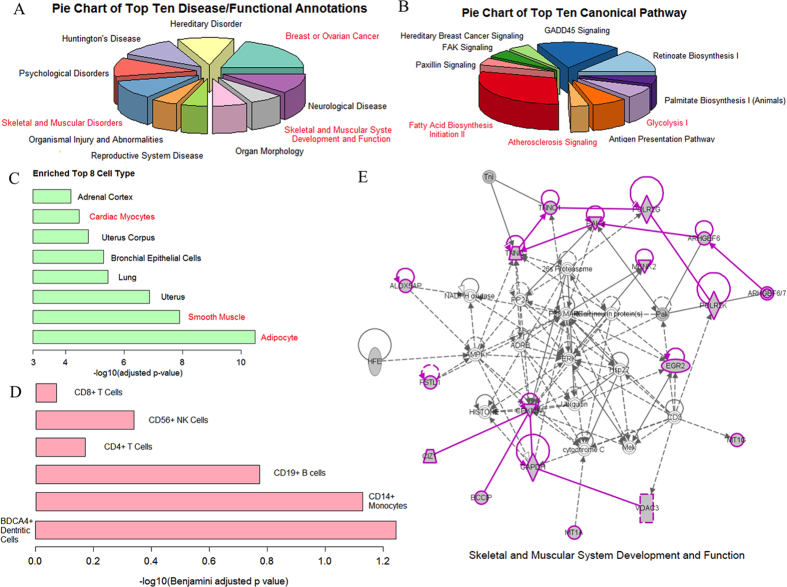
The landscape of ICEGM co-expression gene module (cluster 5). (**A**) Top ten enriched disease/functional annotations linked to our ICEGM gene module. (**B**) Top ten enriched canonical pathways involved by genes in our ICEGM gene module. (**C**) The top eight enriched cell types specifying to the genes in ICEGM gene module. (**D**) Immune cells with high significant scores. (**E**) The number one enriched functional network and the genes in ICEGM gene module are highlighted with purple.

The top ten disease/functional annotations and canonical pathways analyzed using the IPA platform are shown in Fig. 2A,B as pie charts, respectively. The ICEGM co-expression gene module (cluster 5) shows an enriched association with the cancer development and skeletal disorder, as well as the metabolism- and atherosclerosis-associated signaling pathways. The top 8 enriched cell types in the cluster 5 included adipocytes, smooth muscle and cardiac muscle cells (Fig. 2C). In addition, the cluster 5 was also enriched with immune cells, including BDCA4^+^ dendritic cells, CD14^+^ monocytes and CD19^+^ B cells (Fig. 2D). The top one enriched functional network (Fig. 2E) was tightly associated with skeletal and muscular development and function. The genes in this network are either directly or indirectly connected. In conclusion, ICEGM is an effective tool for identifying the co-expression gene modules in multiple tissues. Further analysis of the genes identified using ICEGM in the monkey tissues revealed the connectivity of these genes with the development of diseases.

### The role of inflammation-associated genes in CVD and skeletal disorder disease

**Figure 3:**
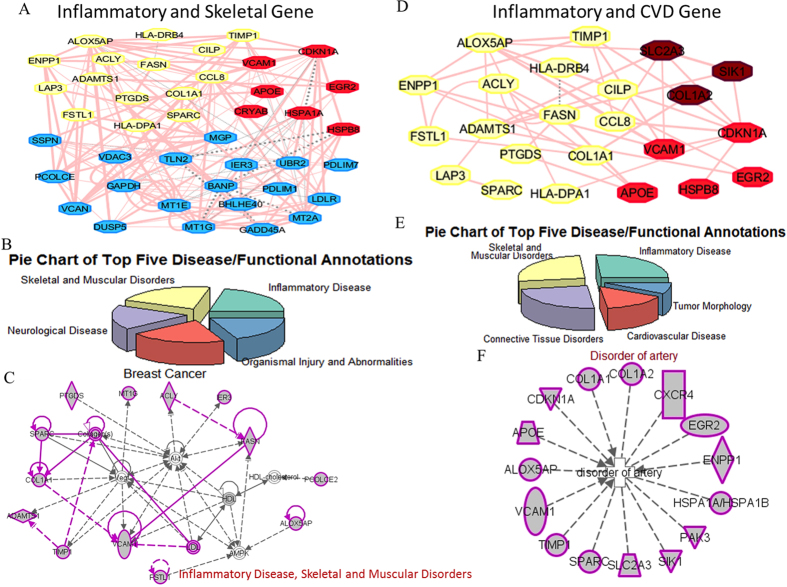
The close connections of inflammation-associated genes with CVD- or skeletal disorder-associated genes. (**A**,**D**) The co-expressed network among the genes associated with inflammation and CVD/skeletal disorder. (**B**,**E**) The top five enriched disease/functional annotations for genes associated with inflammation and CVD/skeletal disorder. (**C**,**F**) The number one enriched functional network for genes associated with inflammation and CVD/skeletal disorder.

The data presented in the Fig. 2 and Figure S2 suggested that the inflammation-related genes appear to associate with skeletal muscle disorder and CVD. To validate that, we first examined the connectivity of inflammation-associated genes with the skeletal muscle disorder- or CVD-associated genes. Figure 3A,D shows the co-expression gene networks for genes associated with inflammation and skeletal muscle disorder/CVD, respectively. The top five disease/functional annotations for inflammation and skeletal muscle disorder/CVD genes are shown as pie charts in Fig. 3B,E, respectively. The functional connectivity of inflammation and skeletal muscle disorder genes is shown in the top rank functional network: inflammation disease, skeletal and muscular disorders (Fig. 3C). The three top-ranked enriched functional/disease annotations obtained from the IPA platform are inflammation, skeletal disorder and muscular disorder (Fig. 3E). The functional connectivity of inflammation and CVD genes is shown in the top rank functional network: disorder of artery (Fig. 3F).

To further validate the connectivity between inflammation, skeletal disorder, and CVD, we assessed the association of these genes with the physical and cardiac function related traits in the monkeys. The monkeys were divided into two groups based on their physical traits (the left top panel of Figure S3). When taking a close look at these two subgroups, we found that the subgroup one were those young monkeys, and subgroup two were those old monkeys, thus we called these two subgroups as young muscle monkey group and old muscle monkey group, respectively. The physical traits included average walking speed (AVE.WALK.SPEED), Body Mass Index (BMI), knees OA grade (osteoarthritis score), VAT and SAT volume and attenuation (density), pericardial fat volume and attenuation, whole body muscle volume scanned by CT (CT.VolumeMuscle), whole body fat volume scanned by CT (CT.VolumeFat), whole body muscle/fat Interface volume canned by CT (CT.VolThreshold), and whole body bone volume scanned by CT (CT. VolumeBone) (left panel of Figure S3). The boxplot describing the changes among young and old monkey groups was drawn in Figure S4. Comparing with the young muscle group, the old muscle monkeys had lower volume and higher density of fat tissues (except for getting lower density of pericardial fat), higher volume of bone and muscle tissues, lower average walk speed and BMI, and higher kness OA grade and muscle/fat Interface volume. The differential expression of those genes associated with inflammation and muscle disorder in the young and old muscle groups are shown in the right top panel of Figure S3. Our analysis indicated that the aged muscle monkeys had higher expression levels of the genes associated with inflammation and muscle disorder. We also divided the monkeys into two groups based on the cardiac traits: normal and risky heart (left bottom panel of Figure S3). The cardiac traits included interventricular septal thickness (IVS), peak strain (ApexPeakStrain), systolic blood pressure (SYS), and diastolic blood pressure (DIA). The risky heart group consisted of the monkeys with unhealthy cardiac traits, such as higher IVS, ApexPeakStrain, SYS, and DIA (Figure S5). The differential expressions of the genes associated with inflammation and CVD in the normal and risky heart group are shown in the right bottom panel of the Figure S3. These data suggested that the monkeys with an increased risk of cardiac disease had a lower expression level of the genes associated with inflammation and CVD. Our results further confirmed the associations between expression of the genes associated with inflammation and muscle/heart disease and the physical/cardiac traits, indicating the inflammation-associated genes play important roles in maintenance of cardiac/physical function through working together with CVD and skeletal disorder-associated genes.

### The coordination of immune, metabolism and atherosclerosis-associated signalling in the adipocytes, immune cells, smooth muscle cells, and cardiomyocytes

**Figure 4:**
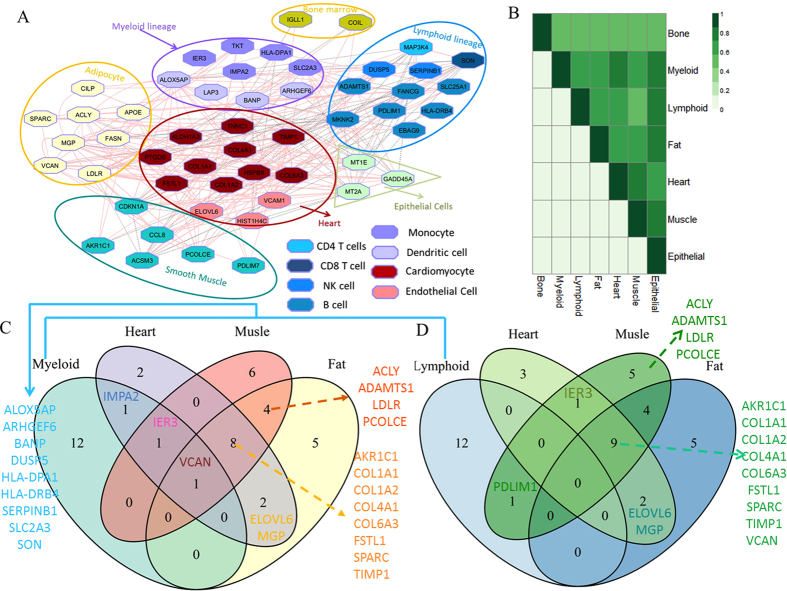
Cell-type enrichment analysis on the genes associated with inflammation, CVD, and skeletal disorder. (**A**) The co-expression network among genes associated with inflammation-, CVD-, and skeletal disorder. Genes were grouped into different cell types they specific to as different colors. (**B**) The correlations among heart, fat, muscle, myeloid, and lymphoid lineage cells. The value of correlation coefficient is shown with the corresponding colors. (**C**,**D**) The Venn diagrams show the overlap genes among heart, fat, muscle, myeloid, and lymphoid lineage cells.

To further illustrate the molecular mechanism, we did cell-type enrichment analysis on those genes associated with inflammation, CVD, and skeletal disorder. As shown in Fig. 4A, these genes were enriched in the adipocytes, smooth muscle cells, cardiomyocyte, myeloid lineage and lymphoid lineage cells. The overlapping genes are shown in Fig. 4C,D. Versican (VCAN), a large extracellular matrix proteoglycan, is shared by heart, fat, muscle, and myeloid lineage cells. The GO annotations related to this gene include calcium ion binding and hyaluronic acid binding. VCAN plays a role in cell adhesion, migration, and proliferation^22^,^23^. Recent studies have associated VCAN with atherosclerosis^24^, vascular disease^25^, inflammation^23^, smooth muscle^26^, and human cancer^27^,^28^. All these results suggested that our ICEGM module could reveal the co-expression genes appeared to play important roles in maintenance the cardiac and physical function in the aging monkeys. There are 10 genes shared by heart, muscle, and fat cell, and nine genes shared by lymphoid and myeloid lineage cells. The over 0.4 correlation coefficients, suggested the close relationship among genes enriched in adipocytes, smooth muscle, cardiomyocyte, and immune cells (Fig. 4B).

**Figure 5:**
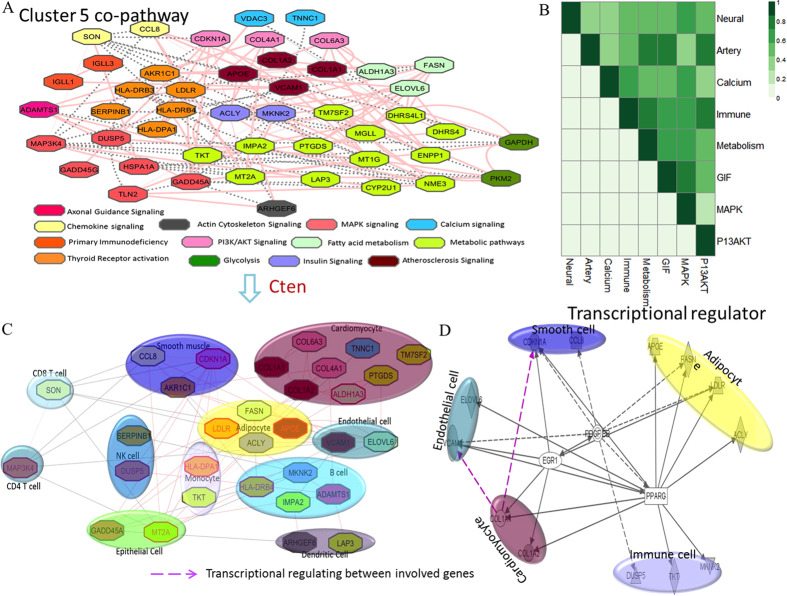
Canonical pathways analysis on the genes associated with inflammation, CVD, and skeletal muscle disorder. (**A**) The co-expression networks for genes associated with inflammatory, CVD, and skeletal disorder. Genes are grouped into diverse pathways they involved in as different colors. (**B**) The correlations among pathways associated with co-expressed inflammatory, CVD, and skeletal disorder genes. The value of correlation coefficient is shown with the corresponding colors. (**C**) The pathways and cell types lined to those co-expressed inflammatory, CVD, and skeletal disorder genes. (**D**) The regulators shared by genes in fat, immune, heart, and muscle cell.

To further understand the relationships among adipocytes, smooth muscle cells, cardiomyocyte, and immune cells following aging. We did canonical pathway analysis on the genes associated with inflammation, CVD, and skeletal muscle disorder. Figure 5A shows that inflammation-, CVD-, and skeletal muscle disorder-associated genes participated in the immune-related (primary immunodeficiency, PI3/Akt signaling, MAPK signaling), metabolic (Glycolysis signaling, Fatty acid metabolism, insulin signaling), neural development (Axonal Guidance signaling), calcium signaling, and atherosclerosis signaling pathways. The correlation coefficients were over 0.4 among those pathways (Fig. 5B), suggesting the close relationship among genes among those pathways. We also combined cell types with the signaling information in Fig. 5C. As shown in Fig. 5C, the coordinated pathways in the individual cells were shared by diverse cells. For example, in the cardiomycytes, metabolic and p13/AKT signaling collaborated with atherosclerosis signaling to maintain cardiac function, while adipocyte, endothelial cell communicates with cardiomyocyte through the atherosclerosis signaling pathway. Furthermore, by introducing regulator information from IPA report, we found the same regulators shared by the genes in smooth muscle cells, adipocytes, immune cells, cardiomyocytes, and endothelial cells, including EGF1, PPAR gamma, PDGFBB (Fig. 5D), indicating close relationships among above cells. In conclusion, we have good reason to speculate that inflammation play an important role in the development of CVD or skeletal muscle disorder during aging through immune-related, metabolic, calcium signaling, and atherosclerosis signaling coordinating in adipocytes, immune cells, smooth muscle cells, and cardiomyocytes.

Early studies have explored the role of inflammation in the development of CVD^29^,^30^,^31^. Berg *et al*. described the possible process about how hypertrophic adipocytes result in atherosclerosis^32^ as follows. In the aging animals, the hypertrophic adipocytes with inefficient metabolism secrete more adipokines and lipoproteins. Increasing of these adipokines, to attract monocytes into the lumen of artery^33^, would lead to the insulin resistance and vasodilation^32^. Meanwhile, increased plasma concentration of adipocyte-derived cholesteryl ester transfer protein (CETP) results in a reduced level of high density lipoprotein (HDL) and increased level of low density lipoprotein (LDL). Once the redundant LDL enters intima and becomes oxidized LDL (OxLDL), it would help the transformation of macrophages into foam cells. The foam cells are the main elements of lipid core. Meanwhile, monocyte chemoattractant protein-1 (MCP-1) would attract smooth muscle cells (SMC) immigrate into intima from the media, leading to plaques prone to rupture with thin fibrous caps, necrotic cores and rich in macrophages. These processes together would lead to the lumen of artery narrow down, and finally result in the atherosclerosis (Figure S6)^33^. The whole process involves immune-related (cytokines involved signalling), metabolic (hypertrophic adipocytes resulting from inefficient metabolism), and atherosclerosis signaling. It also involves adipocytes, immune cells (such as monocyte), and cardiomyocytes. All these results provide evidences that inflammation plays an important role in the development of CVD during aging through multiple signaling regulations coordinated in the adipocytes, immune cells, and cardiomyocytes. The role of inflammation in skeletal disorder was also investigated previously by several groups^34^,^35^. All of these results indicated that ICEGM is an efficiently systematical framework in decoding the co-expression gene modules in multiple tissues for further analysis the role of the specific genes in the development of diseases.

The module cluster 3 is shown in the Figure S7. We did function, pathway, and cell-type analyses on the cluster 3 and found i] the inflammation-, CVD-, and skeletal disorder-associated genes were enriched and ii] involved in the signaling related to immune development, atherosclerosis, and metabolism; and iii] expressed specifically in adipocytes, cardiac myocytes, smooth muscle cells, and immune cells (Figures S8 and S9). These results together indicated the our ICEGM could unveil the co-expression genes involved in inflammation-, CVD-, and skeletal disorder, and those disease related pathways. Further studying the genes in ICGEM co-expression module might provide the great insight into the molecular mechanism of aging effects on fat and muscle tissues in aged animals.

## Discussion

**Figure 6:**
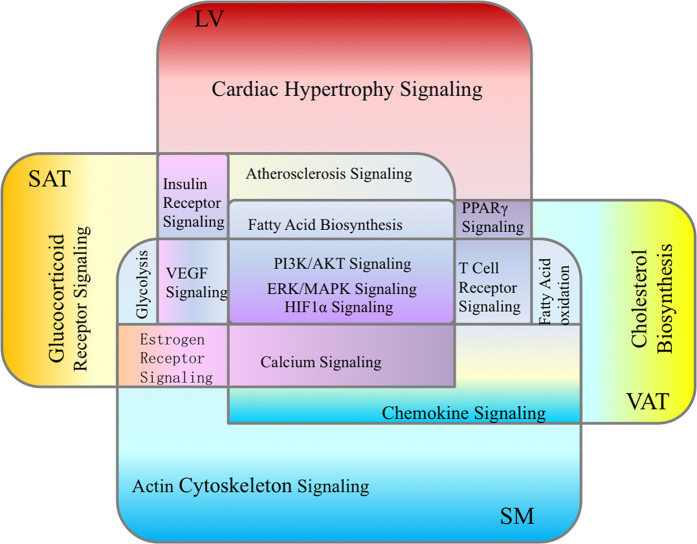
Pathways shared by fat, and heart and skeletal muscle tissues. The pathways in different tissues are shown with different colors. Abbreviation: visceral adipose tissue (VAT), subcutaneous adipose tissue (SAT), left ventricular muscle tissue (LV), and skeletal muscle tissue (SM).

In this study, we developed a novel computational framework, called ICEGM, for identification of co-expression gene modules in multiple tissues. By performing ICEGM on the fat, and heart and skeletal muscle tissues in young and old female vervet monkeys, the common signaling pathways shared by fat, heart and skeletal muscle tissues were unveiled. The metabolic (fatty acid biosynthesis), immune-related and atherosclerosis-associated signalling were shared by fat and heart muscle tissues; while calcium signaling and immune-related pathways were shared by fat and skeletal muscle tissues (Fig. 6). The elucidation of the genes involved in those common pathways might provide the novel therapeutic strategies in the treatment of cardiac and skeletal muscle disorder disease in the aging population. For example, studying the genes involved in common pathways in fat and heart tissues, including PI3K/AKT signaling, ERK/MAPK signaling, and atherosclerosis signaling, may be helpful to identify the therapeutic targets in the treatment of CVD. While the genes involved in common pathways in fat and skeletal muscle tissues, including PI3K/AKT signalling, ERK/MAPK signalling, and calcium signalling, might represent the potential targets in the treatment of skeletal disorder.

Our ICEGM approach simplified the previous HO-GSVD mathematical framework^20^,^21^. Previous HO-GSVD mathematical framework has been used to identify the either ‘common’ or ‘differential’ clusters in multiple datasets. While here, we put more attention on the ‘common’ structures. To this end, the previous framework is simplified to fit the simple case. Specifically, the Gaussian Mixture Model was applied to test the significance of bimodal distribution of right basis vectors, which was calculated by ‘GMMtest’ function in Matlab. And the posterior probability was calculated by the fdrtool function in R package and applied to classify the genes as co-expression genes. Instead of using a complicated mathematical framework for connectivity validation of gene modules, we introduced DAVID bioinformatics resource to evaluate the functional connectivity in our study. As same as previous HO-GSVD mathematical framework in refs 20,21, ICEGM is parameters-free and reproducible, because it didn’t contain the pre-defined parameters and random processing procedure. The number of genes in ICEGM modules was determined through the cut-off of the q-value, which measures the significance of how likely the gene was classified as the co-expression gene. We kept the modules with a relative large number of genes to enhance the interpretation of biological meaning.

The important roles of inflammation in maintaining cardiac and physical function were indicated through comprehensive functional/disease annotation and analysis of the signaling networks and cell enrichment. This is in line with the previous studies showing that the risk of atherosclerotic cardiovascular disease is increased 2- to 3-fold in the type 2 diabetes^36^, obesity contributes to insulin resistance in the type 2 diabetes^37^,^38^, and chronic inflammation play critical roles in obesity and the metabolic syndrome^39^. Early studies found that inflammation represents a key factor contributing to the loss of muscle mass and strength in the elderly^34^,^35^. In conclusion, our findings are consistent with the previous results and also provide a guide for selection of the potential target agents for the treatment of the aging population with CVD or skeletal muscle atrophy. For example, searching the genes involved in common pathways in fat and heart muscle tissues may lead to the potential therapeutic targets for the treatment of CVD. While the genes involved in common pathways in fat and skeletal muscle tissues can be used as potential targets for the treatment of skeletal disorder. Validating above results using *in vitro* and *in vivo* models are needed in the future studies.

In summary, our study indicates that ICEGM is an effective approach for identification of aging-specific co-expression gene modules in monkeys. Decoding those genes associated signaling network, function and cell enrichment is useful for the demonstration of potential mechanisms in the development of specific diseases. To further study the molecular mechanism of CVD and skeletal muscle disease in the old population, incorporation of cardiac and physical traits with the co-expression data in a systematic approach to identify the cardiac and physical traits specific common pathways in fat and muscle tissues, are needed in future work.

Previous studies on aging show that fat, and heart and skeletal muscle are involved in longevity and age-related metabolic dysfunction. For example, in old age, major changes in fat distribution and function are associated with diabetes, hypertension, cancer, cognitive dysfunction, and atherosclerosis^2^,^40^. Skeletal muscle atrophies and declines in force with aging, termed sarcopenia. This condition limits daily living activities and contributes to morbidity and mortality in older adults^4^,^41^,^42^. The aging process contributes to the changes seen in the cardiovascular system in older people^43^. The tissue-specific regulations that are important for tissue identity^44^, and the fat and muscle specific gene modules would help us to assess the effects of age on functional and molecular phenotypes in fat, heart and muscle tissues. It is quite straightforward to hypothesize that functional and molecular phenotypes that vary with aging may be associated with specific gene modules, which consists of a list of genes that display different expression patterns between old and young organisms. Thus, in the future, we would attempt to develop a systematically computational model for detecting the tissue-specific gene module, which not only contains the genes displaying significantly different expression levels in old and young animals, but also associated with aging traits, including body composition, and heart and physical function. This effort will improve our understanding on the effects of age on fat, and heart and skeletal muscle tissue through actions of genes in those modules.

## Materials and Methods

### Animals

The gene expression data were collected from 11 adult female African green vervet monkeys (*Chlorocebus aethiops sabaeus*), including 5 young adults (age 8–10 years, corresponding roughly to human beings in their thirties) and 6 older adults (age 21–26 years, corresponding roughly to human beings in their seventies). All monkeys were born at the Vervet Research Colony at University of California, Los Angeles, and raised in the social groups managed to reflect the natural social composition of vervet groups in the wild. Animals were transferred to the Wake Forest Primate Center in Jan/Feb 2008. All animals had free access to soy protein-based chow and water. All of the animals used in this study were in good health, with normal posture and locomotion patterns, and not pregnant or with offsprings less than 9 months of age. All of the monkeys lived in stable social groups of 11–49 monkeys in housing units with approximately 28 *m*^2^ indoor and 111 *m*^2^ outdoors. They were observed in the outdoor section, which was furnished with perching and climbing structures that enabled all the locomotor behaviors were under surveillance. After behavioral observations completed, the monkeys were lived in pairs and housed in doubled cages for 1 year prior to tissue collection. All procedures involving monkeys were conducted in accordance with state and federal laws and standards of the Department of Health and Human Services. The protocol was approved by the Institutional Animal Care and Use Committee of Wake Forest University.

### Microarray analysis for gene expression

Fat, and heart and skeletal muscle tissues were collected from above monkeys, including visceral adipose (VAT), subcutaneous adipose (SAT), left ventricular muscles (LV), and skeletal muscles (SM). Total RNA samples were extracted using the RNeasy Mini Kit (Qiagen, Valencia, CA). The llumina TotalPrep-96 RNA Amplification Kit (Ambion/Applied Biosystems, Darmstadt, Germany) was used for reverse transcription with 500 ng of total RNA. The Illumina HumanHT-12 v4 Expression BeadChip Arrays were used to perform genome-wide expression analyses. To avoid potential biases due to chip and position, a stratified random sampling technique was used to assign individual samples to specific BeadChips (12 samples/chip) and chip positions.

Pre-processing of data and quality control analyses were done in the open source software R (http://www.r-project.org/) through Bioconductor (http://www.bioconductor.org/) packages. Data were corrected for local background using Illumina’s GenomeStudio. Quality control analyses and bead type summarization (average bead signal for each type after outlier removal) were done using the beadarray package^45^. The detection P-values were computed using the negative controls on the arrays. The ‘neqc’ function of the limma^46^ package was used to estimate the true signals and remove per-array technical effects. After quantile normalization by addition of a recommended (small) offset, log2 transformation, and elimination of control probe data from the normalized expression matrix, we got the total of expressions for 7,680 probes with average detected *p*-value less than 0.01 across all four tissues. The probes were re-annotated to genes through the official annotation files for Illumina HumanHT-12 v4 Expression BeadChip Arrays.

### Data representation

The microarray data from four types of monkey tissues were represented by four large-scale matrices with the columns as genes and rows as samples: 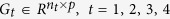, where *n*_*t*_ is the number of samples in *t*th tissue (here *n*_*t*_ = 11 for *t* = 1, 2, 3, 4), and *p* is the number of genes (7,680). We aimed to identify the commonalties from the four matrices through matrix factorization.

### Learning co-expressed gene modules through a simplified mathematic framework of HO-GSVD

HO-GSVD is the upgraded version of Generalized Singular Value Decomposition (GSVD). While GSVD is a generalized version of Singular Value Decomposition (SVD), which extend SVD to two matrices. SVD decomposition of an *n* × *p* real complex matrix *G* is a factorization of the form *G* = *U*Σ*V*^*T*^, where *U* is an *n* × *n* real unitary matrix, Σ is an *n* × *p* rectangular diagonal matrix with non-negative real numbers on the diagonal, and *V* is an *n* × *n* real unitary matrix. The elements of Σ are known as the singular values of matrix *G*. The columns of *U* and *V* are called as the left and right-singular vectors of M, respectively. GSVD is applied into two matrices: 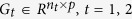, and is given by:


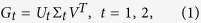


where 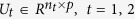, have orthonormal columns, Σ_*t*_ = *diag*(*σ*_*tj*_) ∈ *R*^*p*×*p*^ with 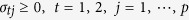, 
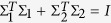
 with *I* as identity matrix. The common factor *V* ∈ *R*^*p*×*p*^ is informative of the cluster structure shared across the two data matrices.

The HO-GSVD was developed for more than two datasets. Under the HO-GSVD framework, matrices 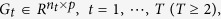 are decomposed as:


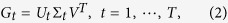


where 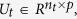 *t* = 1, 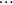, *T*, is composed of normalized left basis vectors, Σ_*t*_ = *diag*(*σ*_*tj*_) ∈ *R*^*p*×*p*^ with 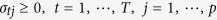, and latent factor matrix *V* ∈ *R*^*p*×*p*^ is composed of normalized right basis vectors *v*_*i*_(*i* = 1, 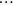, *p*) ∈ *R*^*p*^. The matrix *V* indicates the common structures shared by original datasets. Thus, right basis vectors could allow us to identify a set of genes (gene module) with similar expression pattern across multiple conditions. They were defined as the solution of the eigen-decomposition problem of the matrix^20^,^21^:


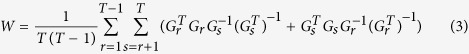


The 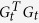 (*t* = 1, 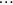, *T*) can be treated as the co-expression data matrix of gene expression, thus when denoted 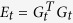 as the co-expression data matrix, then W can be rewritten as:


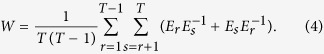


Thus the input for the framework of HO-GSVD could be either expression data or co-expression matrices.

Genes are either co-expression or not, thus the vector *v*_*i*_(*i* = 1, 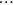, *p*) ∈ *R*^*p*^ with the significant bimodal distribution are helpful to identify the co-expression genes. In another way, the co-expression genes are only taken up a small fraction of the whole gene set, thus the elements in the tail area of the small weight components of bimodal distribution would lead us to the co-expression genes (Figure S1). Based on the above assumptions, the bimodal distribution was tested on each of right basis vectors *v*_*i*_(*i* = 1, 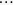, *p*) through Gausssian Mixture Model, calculated by ‘GMMtest’ function in MATLAB, and v^*^ with significant bimodal distribution was chosen to guide us for identification of the co-expression genes. To identify the co-expression genes, the q-value (1-local false discovery rate), which calculated via ‘fdrtool’ R package, was applied here to estimate how likely the elements of v^*^ that were located in the tail area of small weight component of bimodal distribution. The functional enrichment analysis was employed to validate the functional connectivity of ICEGM gene modules through DAVID bioinformatics resources, only gene modules with an enrichment score (Benjamini adjusted p-value) less than 0.05 were kept for further analysis. To evaluate these co-expression gene modules, comprehensive functional/disease annotation, pathway and cell-type enrichment analyses were conducted as follows.

First, we applied IPA platform to perform the functional/disease annotation and canonical pathway enrichment analysis. Then to further validate network properties of ICEGM co-expression gene modules, we did enrichment analyses for the cell-specific gene expression. Cell type enrichment was conducted based on the CTen (Cell Type ENrichment) platform, which identifies changes in the cell-type characteristics of genes based on microarray data. The co-expression networks were drawn through Cytoscape software^47^.

### Calculating the correlation coefficients among different cells and diverse pathways

The correlation coefficients among different cells and pathways were calculated by the average coefficients among genes linked with those cells and pathways. For example, the correlation coefficient between adipocyte and cardiomyocyte was obtained by calculating the average correlation coefficient between genes in adipocyte and genes in cardiomyocyte. The correlation coefficient between metabolism and MAPK signalling was obtained by calculating the average correlation coefficient between genes in metabolism signalling and genes in MAPK signalling.

### The cluster-quality

The cluster-quality for co-expression gene module was defined by the following equation:


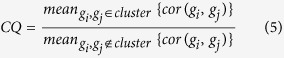


where *cor*(*g*_*i*_, *g*_*j*_) representing the correlation score (such as Pearson’s correlation coefficient) between gene *g*_*i*_ and *g*_*j*_.

## Additional Information

**How to cite this article**: Wang, Y. *et al*. Matrix factorization reveals aging-specific co-expression gene modules in the fat and muscle tissues in nonhuman primates. *Sci. Rep.* **6**, 34335; doi: 10.1038/srep34335 (2016).

## Supplementary Material

Supplementary Information
