# Supplementary material for: Matrix factorization reveals aging-specific co-expression gene modules in the fat and muscle tissues in nonhuman primates
Source: Sci Rep. 2016 Oct 5;6:34335. doi: 10.1038/srep34335 (PMC5050522; doi:10.1038/srep34335)
Supplement: Supplementary Information [file srep34335-s1.pdf]

Supplementary Information

Matrix factorization reveals aging-specific co-expression gene modules in nonhuman primate fat and muscle tissues

Yongcui Wang[1,2], Weiling Zhao[1], Xiaobo Zhou[1*]

1, Department of Radiology, Wake Forest School of Medicine, Winston-Salem, NC, USA.

2, Key Laboratory of Adaptation and Evolution of Plateau Biota, Northwest Institute of Plateau Biology, Chinese Academy of Sciences, Xining, China .

3, Department of Pathology, Section on Comparative Medicine, Wake Forest School of Medicine, Winston-Salem, NC, USA.

*, to whom correspondence should be addressed.

Legend

Table S1. The statistics of ICEGM Gene modules.

Figure S1. Cluster quality for co-expression gene module 3 and 5.

Figure S2. The co-expression network for cluster 5.

Figure S3. The associations between inflammatory genes and cardiac/physical traits.

Figure S4. Boxplot for variations of physical traits.

Figure S5. Boxplot for variations of cardiac traits.

Figure S6. Hypothetical conclusion on the effect of adipose tissue on atherosclerosis.

Figure S7. Co-expression network of cluster 3.

Figure S8. The top enriched functional/disease annotations, canonical pathways, and cell-types for cluster 3.

Figure S9. The enriched cell types with high enrichment score (–log 10 adjusted Benjamini-Hochberg p-value) in cluster 3.

Table S1.

	Q-value cut-off	No. of Genes	DAVID Checking
v_1	0.2	0	×
v_2	0.2	36	×
v_3	0.2	28	√
v_4	0.01	105	×
v_5	0.01	142	√

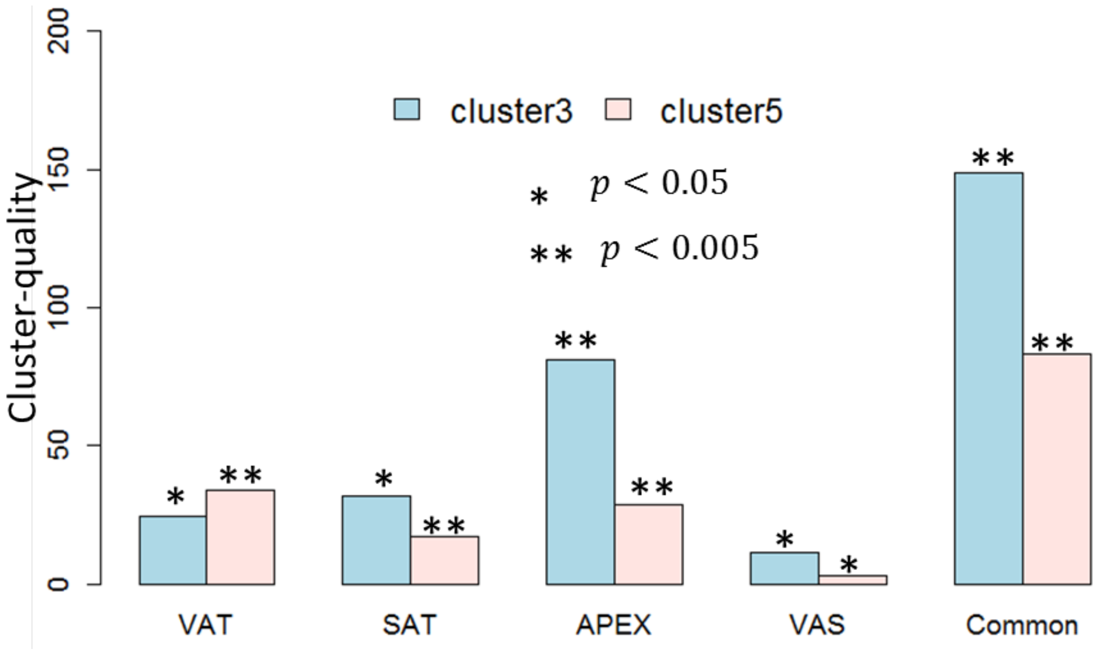

Figure S1. The cluster quality for two functional enriched co-expression gene

module.

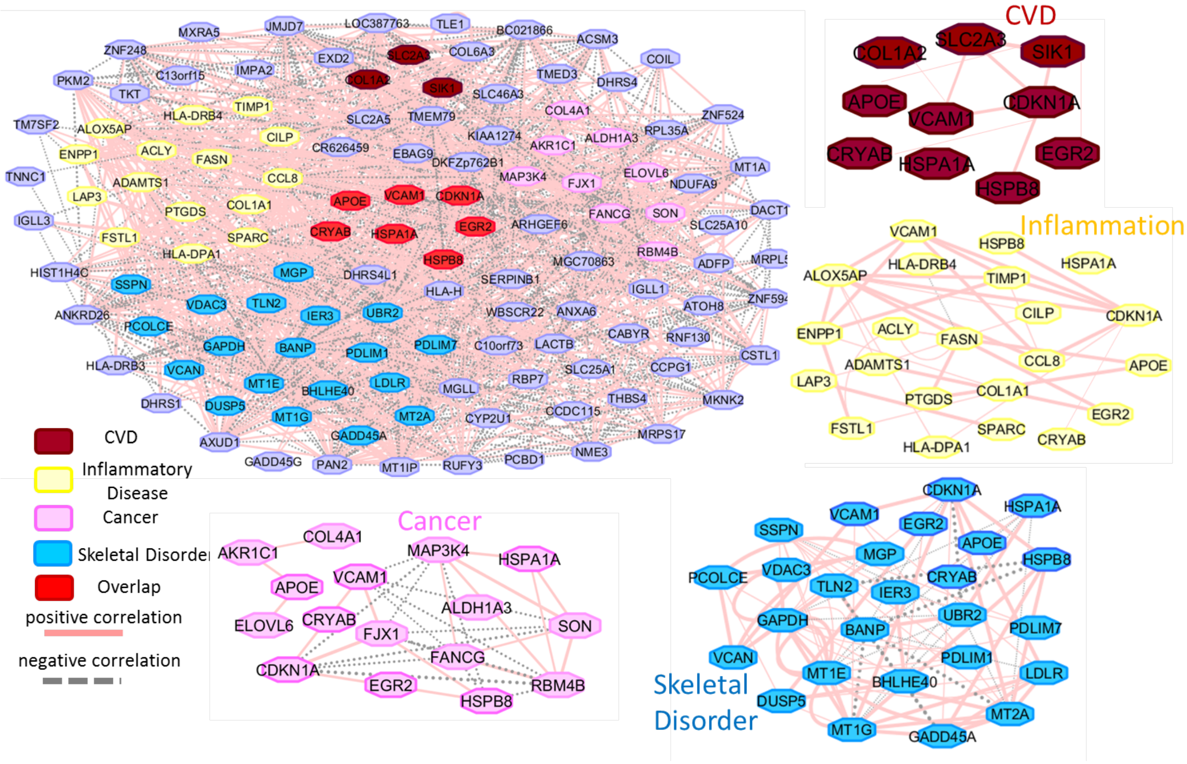

Figure S2. Co-expression networks of the cluster 5. the solid line represents a positive correlation, the dash line represents a negative correlation, and the thickness of a line is proportional to the average covariance value. Only interactions with a absolute covariance value larger than 0.5 are shown here.

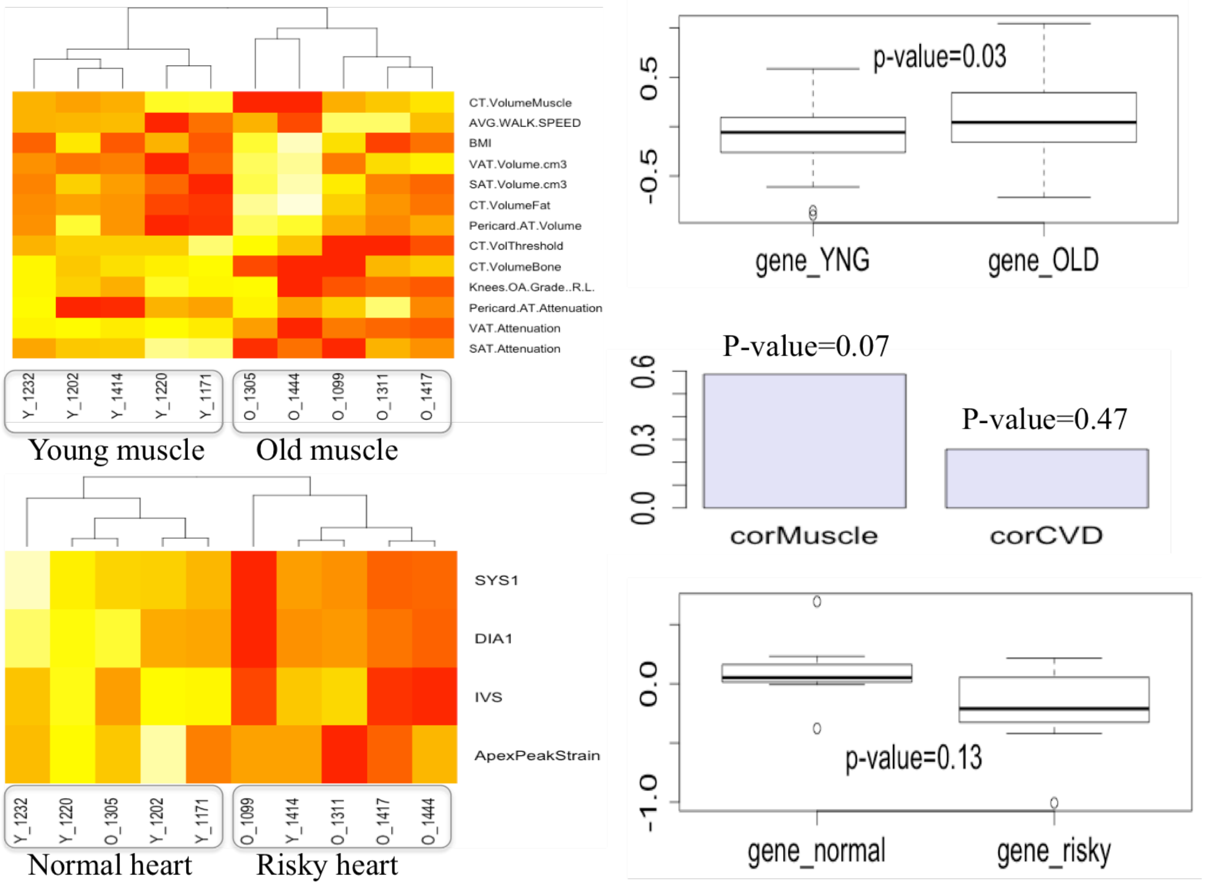

Figure S3. The association of inflammation-, skeletal disorder-, and CVD-associated genes with the physical and cardiac function-related traits in the monkeys. Left panel shows the cluster results based on physical (up) and cardiac (bottom) traits, and the right panel shows the differential expression of the inflammation- and CVD/muscle disorder-associated genes in two monkey groups. The correlation coefficients between expression of the genes and CVD/physical traits are shown in the middle-right panel.

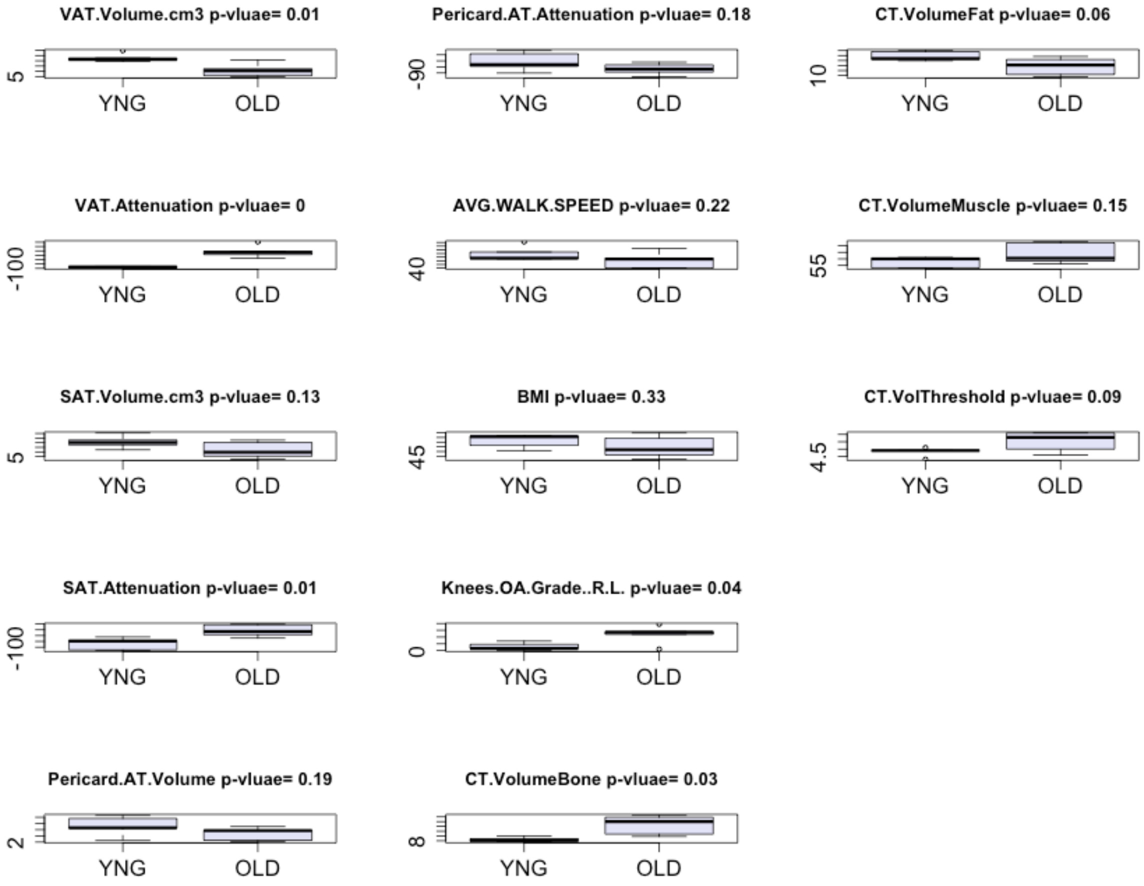

Figure S4. The variations of physical traits among young and old monkeys. It shows that comparing with young group, old monkeys have lower volume and higher density of fat tissues (except for getting lower density of pericardial fat), higher volume of bone and muscle tissues, lower average walk speed and BMI, and higher kness OA grade and muscle/fat Interface volume. The significant variant traits are VAT volume and attenuation (density), SAT volume, Knees OA grade, and Bone volume (with p-value less than 0.05).

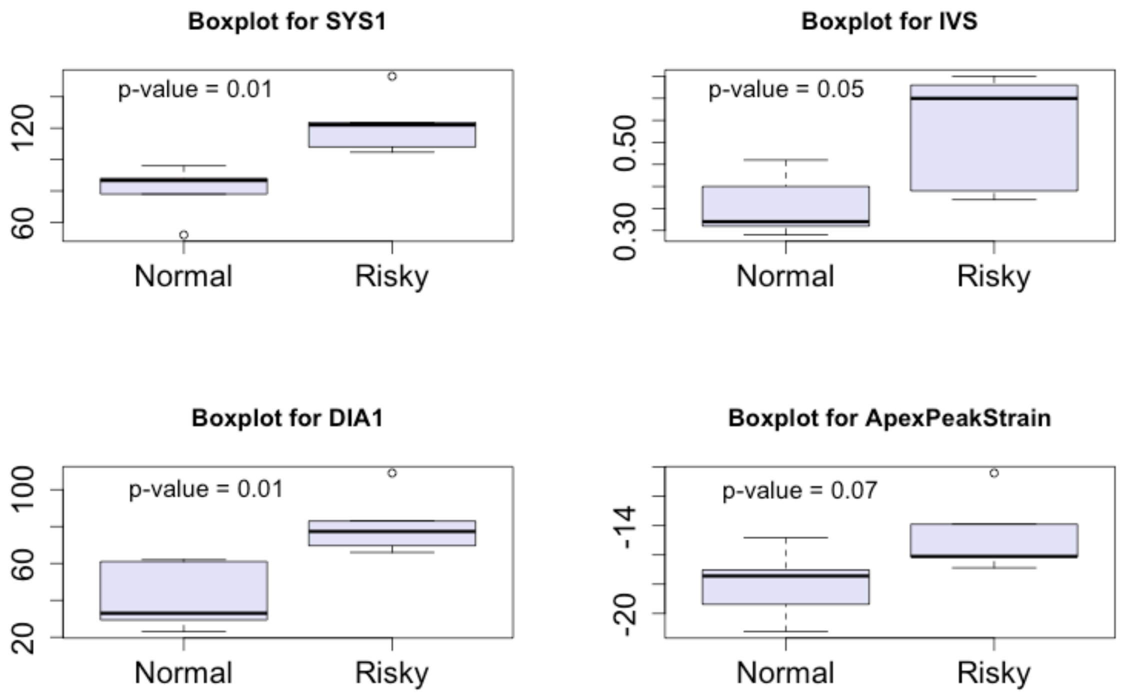

Figure S5. The variations of cardiac traits between monkeys with normal and risky hearts.

Adipocyte dysfunction in atherosclerosis

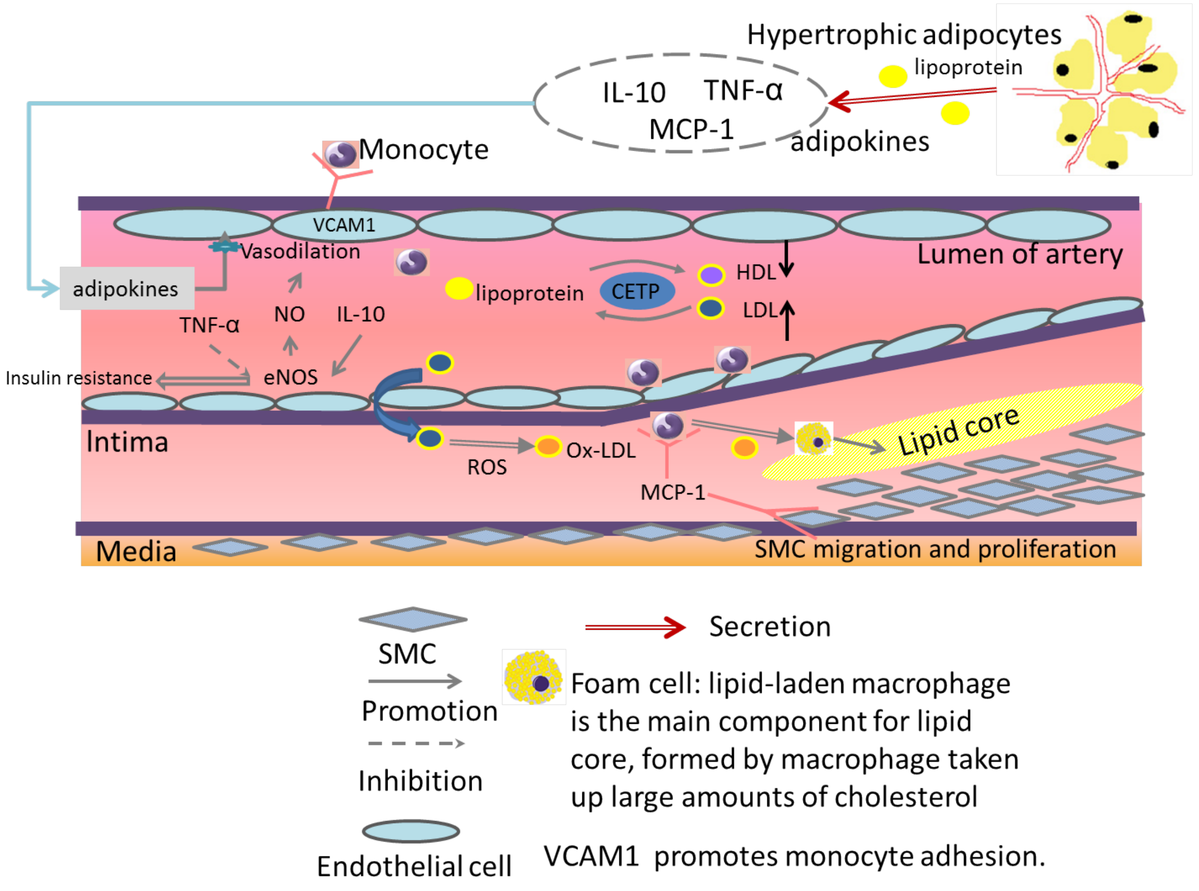

Figure S6. Adipocyte dysfunction leads to atherosclerosis. In the aged animals, dysfunctional adipocyte causing by inefficient metabolism will release adipokines and lipoproteins. Increasing of these adipokines, such as tumor necrosis factor-α (TNF-α), lead to insulin resistance and vasodilation, which attract monocyte to travese endothelial cells and into the lumen of artery. Meanwhile, increased adipocyte-derived cholesteryl ester transfer protein (CETP) plasma concentrations lead to a reduced level of high density lipoprotein (HDL) and an increased number of low density lipoprotein (LDL). Once redundant LDL went into intima and became oxidized LDL (OxLDL), it would help the transformation of macrophages into foam cells. The foam cells are the main elements of lipid cores. Monocyte chemoattractant protein-1 (MCP-1) will attract smooth muscle cells (SMC) to immigrate to intima from the media, leading to plaques prone to rupture with thin fibrous caps, necrotic cores and rich in macrophages. These processes together can make the lumen of artery to narrow down, and finally result in the atherosclerosis.

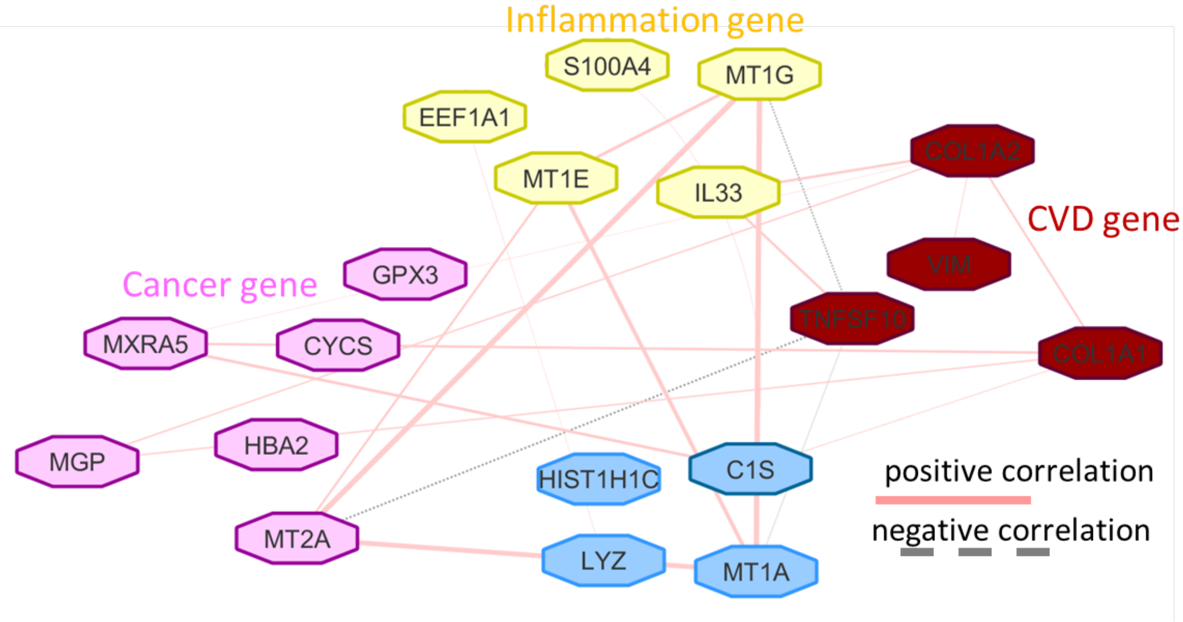

Figure S7. Co-expression network of cluster 3.

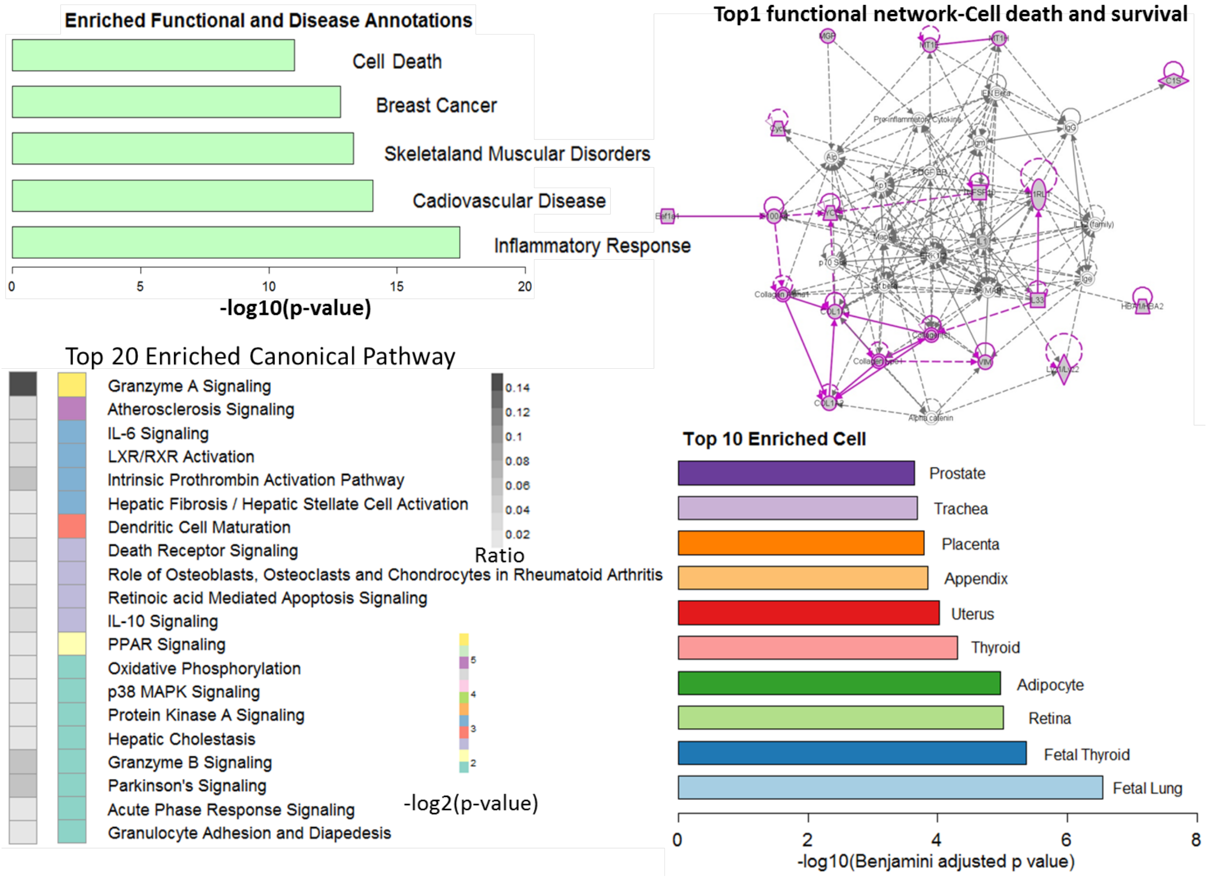

Figure S8. The top enriched functional/disease annotations, canonical pathways, and cell-types for the cluster 3.

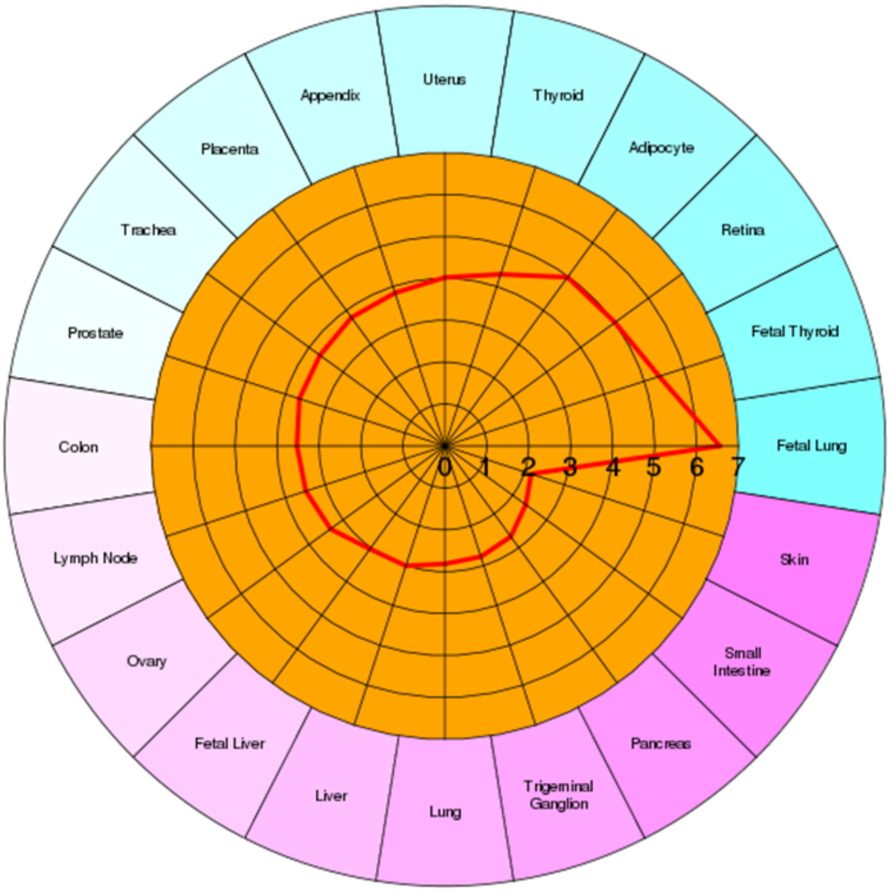

Cardiac Myocytes: 2.091 Bone Marrow:1.3221
BDCA4+ Dentritic Cells: 0.392 CD14+ Monocytes:0.971
Smooth Muscle:1.419

Figure S9. The enriched cell types with high –log 10 adjusted Benjamini-Hochberg p-value in the cluster 3.
